# Cell population balance of cardiovascular spheroids derived from human induced pluripotent stem cells

**DOI:** 10.1038/s41598-018-37686-1

**Published:** 2019-02-04

**Authors:** Yuanwei Yan, Julie Bejoy, Junfei Xia, Kyle Griffin, Jingjiao Guan, Yan Li

**Affiliations:** 1 Department of Chemical and Biomedical Engineering, FAMU-FSU College of Engineering, Florida State University, Tallahassee, FL USA; 2 Present Address: Waisman Center, University of Wisconsin-Madison, Madison, WI USA; 3 Present Address: Department of Bioengineering, College of Engineering, Northeastern University, Boston, Massachusetts 02115 USA

## Abstract

Stem cell-derived cardiomyocytes and vascular cells can be used for a variety of applications such as studying human heart development and modelling human disease in culture. In particular, protocols based on modulation of Wnt signaling were able to produce high quality of cardiomyocytes or vascular cells from human pluripotent stem cells (hPSCs). However, the mechanism behind the development of 3D cardiovascular spheroids into either vascular or cardiac cells has not been well explored. Hippo/Yes-associated protein (YAP) signaling plays important roles in the regulation of organogenesis, but its impact on cardiovascular differentiation has been less evaluated. In this study, the effects of seeding density and a change in YAP signaling on 3D cardiovascular spheroids patterning from hPSCs were evaluated. Compared to 2D culture, 3D cardiovascular spheroids exhibited higher levels of sarcomeric striations and higher length-to-width ratios of α-actinin^+^ cells. The spheroids with high seeding density exhibited more α-actinin^+^ cells and less nuclear YAP expression. The 3D cardiovascular spheroids were also treated with different small molecules, including Rho kinase inhibitor (Y27632), Cytochalasin D, Dasatinib, and Lysophosphatidic acid to modulate YAP localization. Nuclear YAP inhibition resulted in lower expression of active β-catenin, vascular marker, and MRTF, the transcription factor mediated by RhoGTPases. Y27632 also promoted the gene expression of MMP-2/-3 (matrix remodeling) and Notch-1 (Notch signaling). These results should help our understanding of the underlying effects for the efficient patterning of cardiovascular spheroids after mesoderm formation from hPSCs.

## Introduction

Human pluripotent stem cells (hPSCs) are promising sources to generate human cardiovascular progenitors and cardiomyocytes for transplantation and drug toxicity study, because of the difficulty in obtaining primary human cardiomyocytes and their reduced proliferation in culture^1–10^. Highly pure cardiomyocytes can be generated from hPSCs by modulating bone morphogenetic proteins (BMP) or Wnt family proteins in 2D cultures^11–14^. Wnt signaling has a biphasic effect on cardiac tissue development, where early Wnt activation enhances mesoderm induction, at late stage Wnt signaling needs to be suppressed for cardiac differentiation^12,13,15^. In order to mature cardiomyocytes and enable scalable production, spheroids of cardiac cells or the differentiated progenitors from three-dimensional (3D) undifferentiated hPSC aggregates have been generated^1,16–20^. Compare to 2D cultures, 3D spheroid cultures better recapitulate biological features of human cardiovascular tissues and more accurately mimic early-development of the heart with distinct spatial organization, for example, the 3D systems promote sarcomeric striation of cardiac muscle cells and metabolic maturation^16–19^. Moreover, microparticles or nanowires can be added into 3D spheroids to achieve localized delivery and electrical stimulation^17,21,22^.

The 3D spheroid cultures can be heterogeneous. Cardiac organoids have been recently reported with the spheroid formation by mixing hPSC-derived cardiomyocytes, cardiac fibroblasts, and human umbilical vein endothelial cells (3:6:1), or through micropatterned substrates^23,24^. The formed cardiac organoids have lumenized vascular network in the developing myocardium and respond to pharmacological compounds^23^. Vascularization of cardiac tissues was also investigated using human cardiac microvascular endothelial cells^25^. Transplantation of hPSC-derived cardiomyocytes, endothelial cells, and smooth muscle cells showed much better cell engraftment than cardiomyocytes alone in a large animal model^26,27^. 3D cardiovascular spheroids promote cell-cell and cell-matrix interactions and can be patterned into cardiac cells or vascular cells depending on the culture parameters such as cell density, medium components, and substrate compliance^28–30^. Among these, cell density must be optimized for cardiovascular lineage specification.

One signaling event that is influenced by cell density is Hippo/Yes-associated protein (YAP) signaling^31^. Hippo/YAP signaling plays important roles in the regulation of heart size and shapes during organogenesis^32,33^ and in promoting cardiac regeneration^33,34^. Activated Hippo pathway leads to phosphorylation and inactivation of YAP as well as its degradation. When Hippo is inhibited, the YAP is activated and transported to the nucleus. Hence the shuttling of YAP affects proliferation and commitment of cardiac progenitors^35^. For example, YAP was found to co-localize with the early cardiac transcription factor GATA-4^35^. YAP also regulates insulin-like growth factor signaling and thereby controls cardiomyocyte proliferation and embryonic heart size^36^. YAP/TAZ silencing in cardiac progenitors results in up-regulation of endothelial-specific genes whereas YAP/TAZ activation results in upregulation of cardiomyocyte genes^35^. YAP localization is affected by cell density^31^, Wnt signaling^37,38^, the Rho signaling, and actin cytoskeleton (stress fibers) polymerization^39^. However, how these signaling pathways interplay during cardiovascular patterning from hPSCs is not well studied.

The objective of this study is to investigate the balance of cardiac and vascular populations derived from human induced pluripotent stem cells (hiPSCs) by modulation of cell density and YAP localization in 3D spheroid cultures toward the long-term goal of generating cardiovascular tissues or organoids^40^. (1) Cardiac differentiations from hiPSCs in 3D aggregates were performed and characterized in comparison to 2D differentiation. (2) The cell seeding density of 1–5 × 10^4^ cells per spheroid in each well of 96-well plate was evaluated for cellular optimization. (3) Due to the contractility effects in 3D spheroids and the possible involvement of Hippo/YAP signaling^41^, the derived cardiovascular spheroids were treated to redistribute YAP localization, using Rho kinase (ROCK) inhibitor Y27632, Cytochalasin D, Dasatinib, and Lysophosphatidic acid (LPA). Active β-catenin expression, MMP-2/3, Notch-1 (for cell-cell communication), MRTF, the transcription factor mediated by RhoGTPases, and the downstream YAP target gene CTGF were analyzed^42,43^. This study should be helpful to generate cardiovascular spheroids of balanced vascular and cardiac cells for disease modeling, drug screening, and cell therapy^24^.

## Results

### Cardiomyocyte differentiation and cardiac spheroid formation

**Figure 1:**
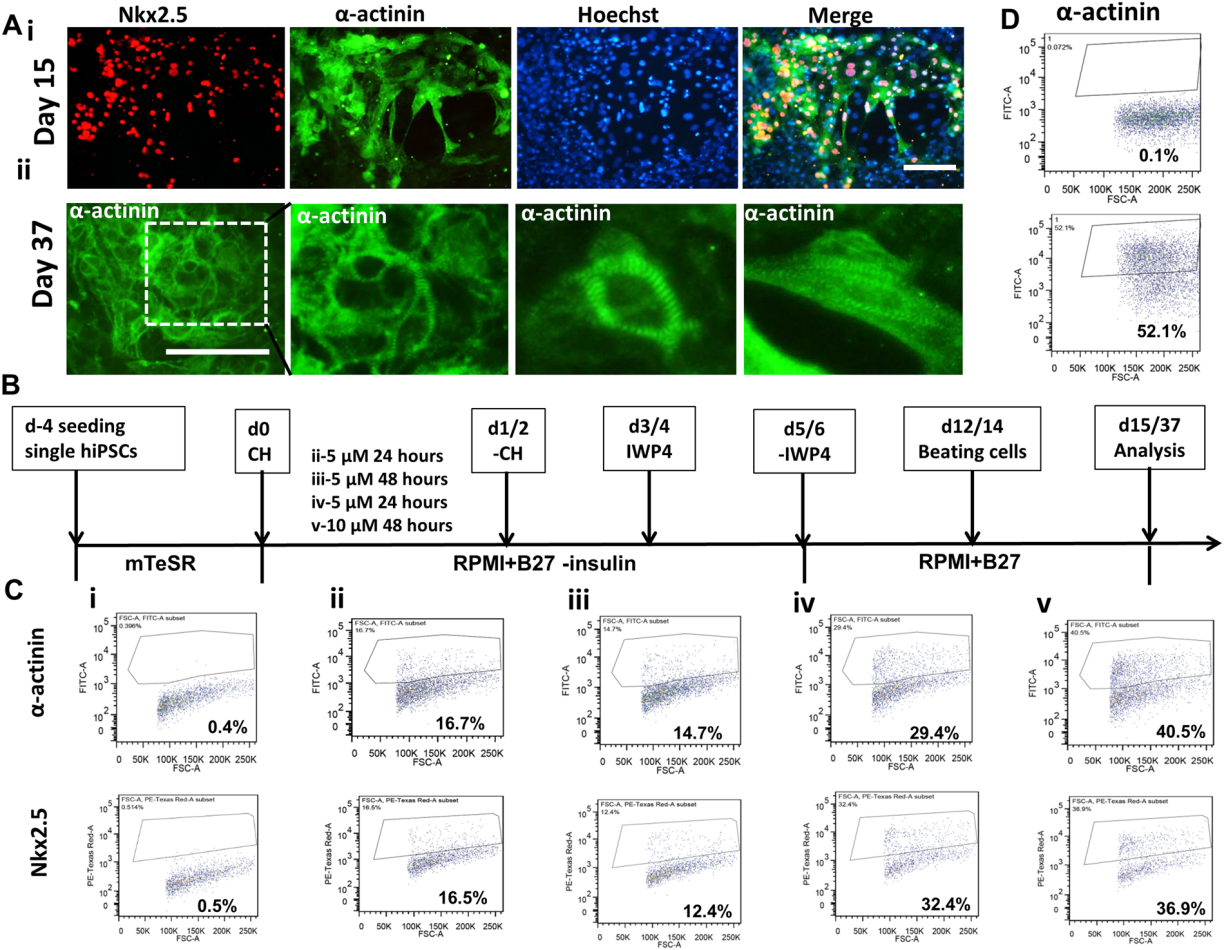
Cardiac differentiation from hiPSCs on monolayer culture (2D). (**A**) (i) Representative fluorescent images of cardiac markers: Nkx2.5, α-actinin for cells at day 15; (ii) Representative fluorescent images of cardiac marker- α-actinin for cells at day 37. Scale bar: 100 μm. (**B**) A schematic differentiation timeline using CHIR99021 (CH) and IWP4. (**C**) The expression of cardiac markers Nkx2.5 and α-actinin for CHIR99021 treatment conditions analyzed by flow cytometry: isotypes (i), 5 µM 24 hours (ii), 5 µM 48 hours (iii), 10 µM 24 hours (iv) and 10 µM 48 hours (v) for samples at day 13. (**D**) Activin A/BMP-4 induced differentiation (day 18): Isotype control and α-actinin expression.

Monolayer-based cardiomyocyte differentiation from human iPSK3 cells was preformed using Wnt activation/inhibition method (Supplementary Fig. S1). Generally, the cells started the beating around day 10 and more contraction was observed by day 15 (Supplementary Movie S1). The expression of cardiac markers Nkx2.5 and α-actinin was assessed (Fig. 1). At day 15, a majority of cells showed positive expression of Nkx2.5 and α-actinin (Fig. 1Ai). The sarcomeric structures could be clearly observed at day 37 through staining of α-actinin (Fig. 1Aii). Since the CHIR99021 induction is the critical step for hiPSC-derived cardiomyocyte differentiation, CHIR99021 treatments were optimized based on the expression levels of Nkx2.5 and α-actinin (Fig. 1Bi–v). The flow cytometry data showed that the treatment of 10 µM CHIR99021 for 48 hours had the highest expression of Nkx2.5 and α-actinin (36.9% and 40.5%). So this condition was chosen for the following experiments. The A100/B10 protocol^7,14^, in which cells were sequentially treated with Activin A and BMP4, was also evaluated (Supplementary Fig. S1). At day 18, the expression of α-actinin was 52.1% (Fig. 1C). Taken together, the cardiomyocytes could be generated by Wnt activation/inhibition (Giwi protocol) and A100/B10 protocol from human iPSK3 cells in 2D cultures.

**Figure 2:**
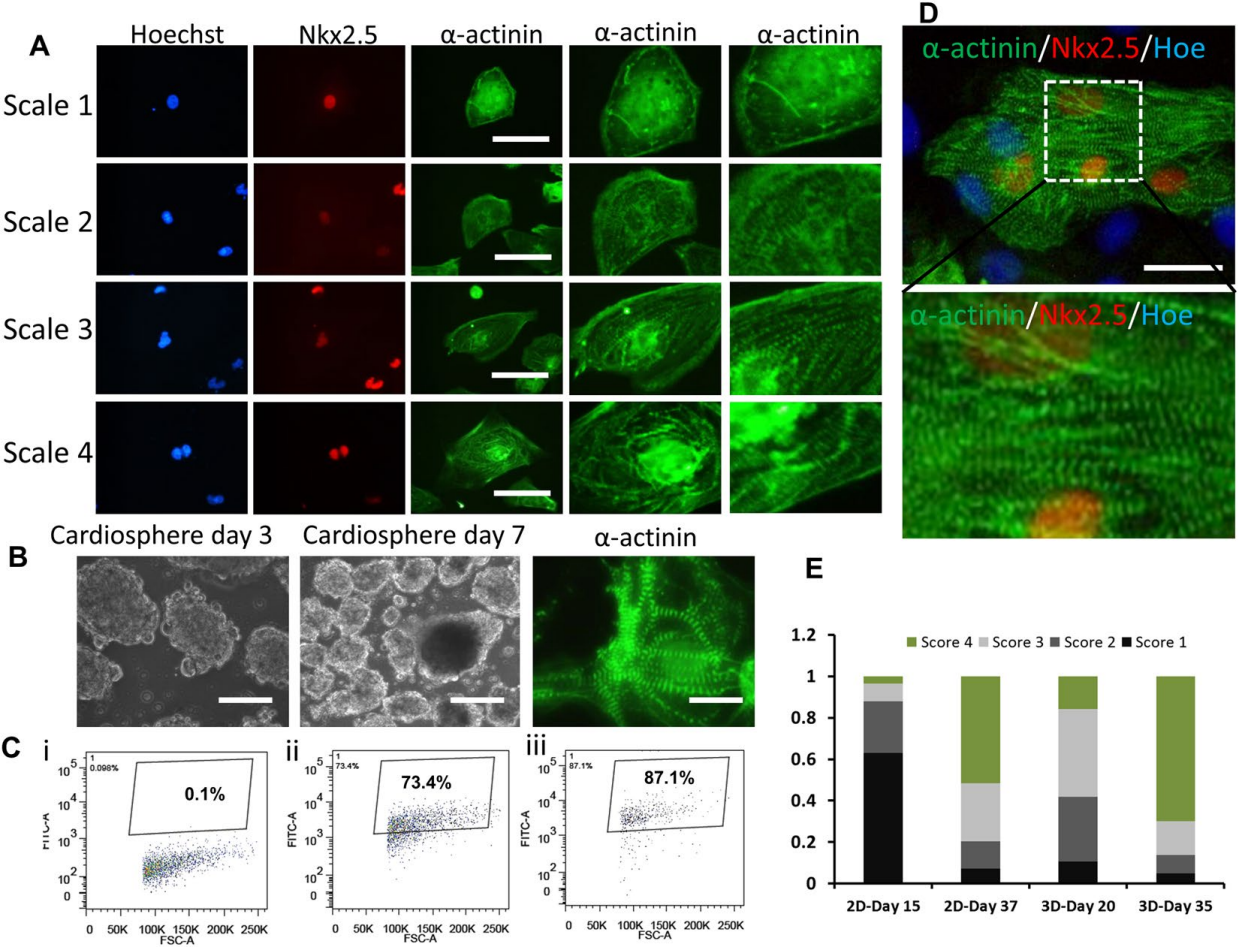
Cardiac spheroid formation and maturation from hiPSCs in 3D culture. (**A**) The level of sarcomeric striations was visualized from high-magnification images and given a score of 1–4. Cells that received a score of 1 stained positively for α-actinin, but without clear sarcomeric striations. Cells that were scored 2,3, or 4 had detectable sarcomeric striations at increasing levels. Scale bar: 50 μm. (**B**) The phase contrast images of cardiac spheres at day 3 and day 7 after initial formation at day 15, and representative fluorescent images of cardiac marker-α-actinin for cardiac spheroids at day 35. (**C**) The expression of α-actinin of 3D samples: isotype (i), parallel 2D (ii), and 3D (iii) by flow cytometry. (**D**) Representative fluorescent image and the enlarged image of cardiac markers: Nkx2.5 and α-actinin for 3D samples at day 35. Scale bar: 100 μm. Hoe: Hoechst 33342. (**E**) Percentage of cells by the scores. Note that cells from 3D cultures displayed higher numbers of cells with more clear sarcomeric striations compared with cells from 2D cultures.

**Figure 3:**
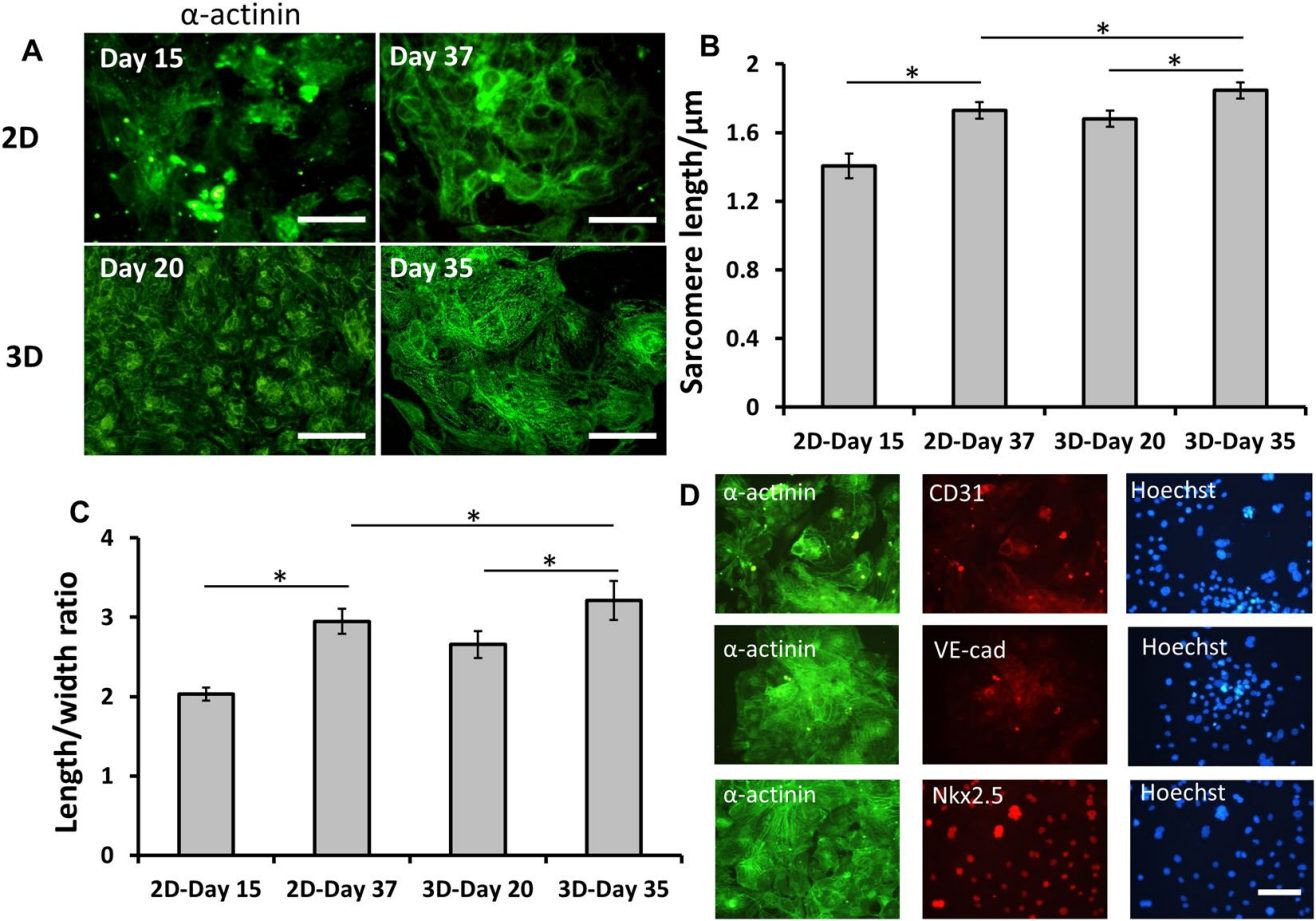
Measurements of sarcomeric lengths and morphology of cardiomyocytes. (**A**) Representative fluorescent image of cardiac marker α-actinin for 2D and 3D cultures. Scale bar: 100 μm. (**B**) The sarcomere length of cells for different samples. (**C**) The length/width ratio of cells for different samples. (**D**) Representative fluorescent image and enlarge image of cardiac markers Nkx2.5, α-actinin and vascular markers CD31 and VE-cadherin for 3D samples at day 35. Scale bar: 100 μm. *Indicates *p* < 0.05.

To enhance the cardiomyocyte maturation, the day 15 cells in 2D monolayer culture were dissociated and seeded in Ultra-Low Attachment (ULA) 24-well plate (Fig. 2). The cells aggregated to form 3D cardiospheres (Fig. 2B), and most of the cardiospheres showed continuously beating (Supplementary Movie S2). The cardiomyocytes from cardiac spheroids showed positive expression of Nkx2.5 and α-actinin (Fig. 2D and Supplementary Fig. S2). At day 35, the expression level of α-actinin for 3D cardiospheres was higher than that of cells from 2D culture (87.1% vs. 73.4%) (Fig. 2C). To compare the maturation of cardiomyocytes, z-line sarcomere organization of cardiomyocytes was analyzed (Fig. 2A). The maturation was determined by the scores of the relative levels of z-line sarcomere organization^18^. Compared to 2D culture, 3D cardiospheres exhibited higher levels of sarcomeric striations (more level-4 cells) (Fig. 2E). Cells from 3D spheres also showed longer sarcomeric structures and higher length-to-width ratios of α-actinin^+^ cells (Fig. 3A–C). Besides, the vascular markers CD31 and VE-cadherin were found in 3D cardiospheres (Fig. 3D), indicating the existence of vascular cells in 3D cardiac spheroids. So our data suggest that 3D cardiospheres cultures are permissive to hiPSC-derived cardiomyocyte maturation.

### Cell density effects on cardiovascular spheroid formation

**Figure 4:**
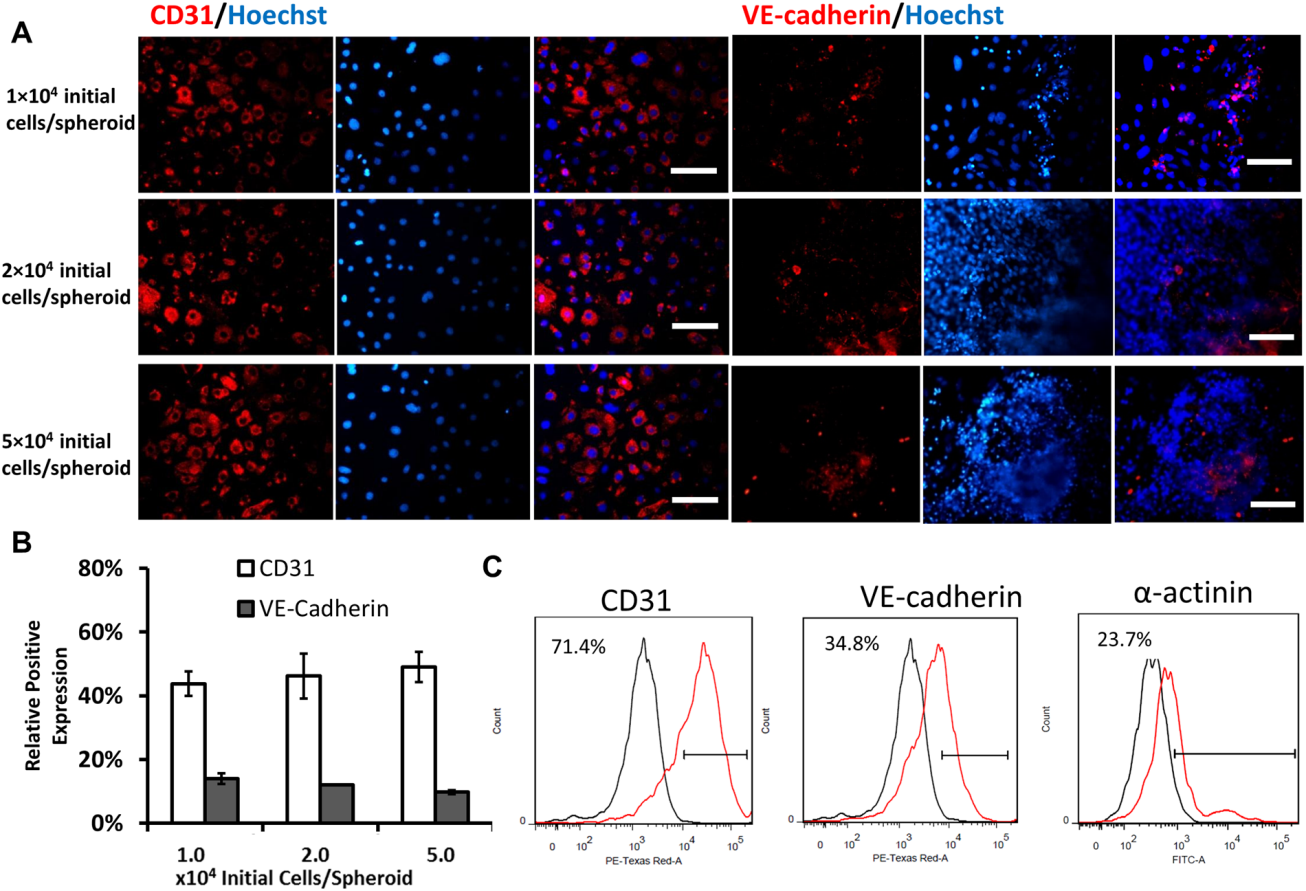
Cell density effects on cardiovascular spheroid formation from hiPSCs. (**A**) Representative fluorescent images of cardiovascular markers CD31 and VE-cadherin for different seeding densities at day 22. Scale bar: 100 μm. (**B**) The quantitative analysis of CD31 and VE-cadherin positive cells (by ImageJ analysis). (**C**) The representative expression of CD31, VE-cadherin and α-actinin by flow cytometry. Black line: negative control; Red line: marker of interest.

**Figure 5:**
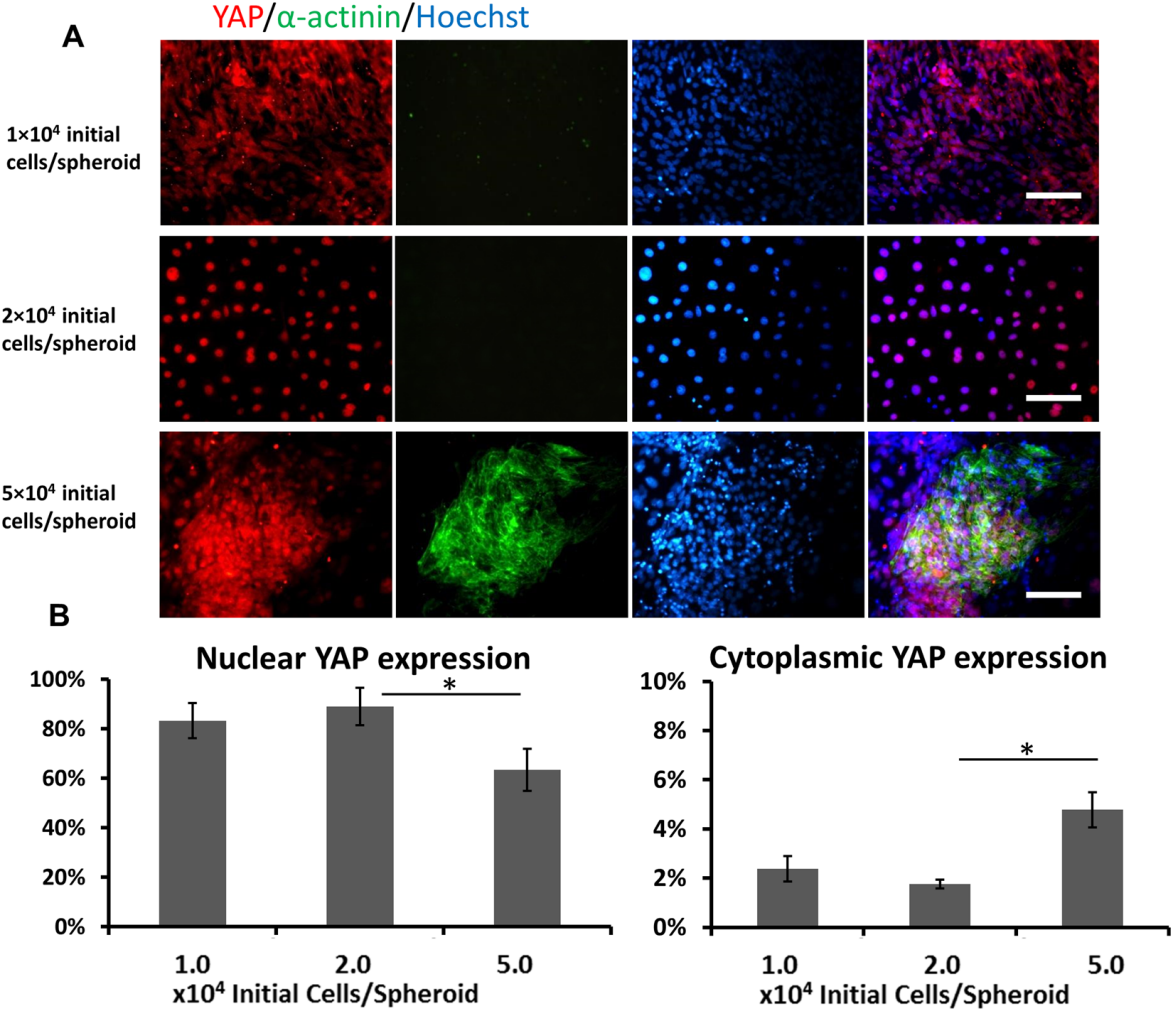
Cell density effects on YAP expression from hiPSCs. (**A**) Representative fluorescent images of markers α-actinin and YAP for different seeding densities (1 × 10^4^, 2 × 10^4^, 5 × 10^4^ initial cells/spheroid in each well of 96-well plate) at day 18. Scale bar: 100 μm. (**B**) The quantitative analysis of nuclear and cytoplasmic YAP expression. *Indicates *p* < 0.05.

The optimized Giwi protocol was translated to a 3D format by generating cardiovascular spheroids in suspension cultures (Fig. 4). Human iPSK3 cells were seeded into ULA 24-well or 96-well plates to form aggregates and then sequentially treated with CHIR99021 and IWP4. Similar to 2D culture, the spheres in suspension were observed to start beating around day 10. The mean beating frequency increased from day 10 to 31 (e.g., 18 to 36 times in one minute) and the number of beating spheres gradually increased over time (Supplementary Fig. S3). The density effects on cardiovascular spheroid formation were investigated by seeding different cell numbers of hiPSCs (1, 2, 5 × 10^4^ initial cells/spheroid) with one spheroid in each well of ULA 96-well plates. At the beginning, cells formed a regular sphere and the cells with higher cell density formed a larger sphere. However, the cardiovascular spheroids were re-organized around day 13 and formed irregular shape (Supplementary Fig. S4). The spheres reached the maximum size around 1500 µM in diameter at day 18. The average size of spheroids for different seeding densities was comparable at a later stage. The expression of cardiac and vascular markers was characterized (Fig. 4A). Cells with three seeding densities exhibited a high expression level of CD31 but low level of VE-cadherin (Fig. 4B). However, cells at 1 and 2 × 10^4^ initial cells/spheroid nearly showed no expression of cardiac marker α-actinin (Fig. 5A). The expression level of α-actinin for 5 × 10^4^ initial cells/spheroid was 23.7% by flow cytometry (Fig. 4C). The localization of CD31 in spheroids was examined using confocal microscopy (Supplementary Fig. S5). CD31^+^ cells were observed inside the spheroids, but not necessarily nearer the periphery of the spheroids. The distribution was not homogeneous but rather localized at some spots.

To interpret the seeding density-dependent balance of cardiac and vascular populations, the role of YAP pathway was evaluated (Fig. 5). It was found that cells of seeding density at 5 × 10^4^ initial cells/spheroid showed less nuclear YAP expression but more cytoplasmic localization (Fig. 5B). Thus, these observations demonstrate that seeding density could influence the balance of cardiac and vascular populations through regulating the YAP signaling.

To evaluate the effects of Geltrex and ROCK inhibitor Y27632 on cardiovascular spheroid formation, cells were cultivated in 1% or 10% Geltrex, and with or without 10 µM Y27632 for 7 days in the ULA 24-well plates (Supplementary Fig. S6). Cells with 1% Geltrex showed good sphere formation and high cell viability while cells with 10% Geltrex did not form spheres at all. For Y27632 treatment, no significant difference was observed compared to the cells without treatment.

### Effects of Cyto D, Y27632, Dasatinib, and LPA on cardiovascular spheroid patterning

**Figure 6:**
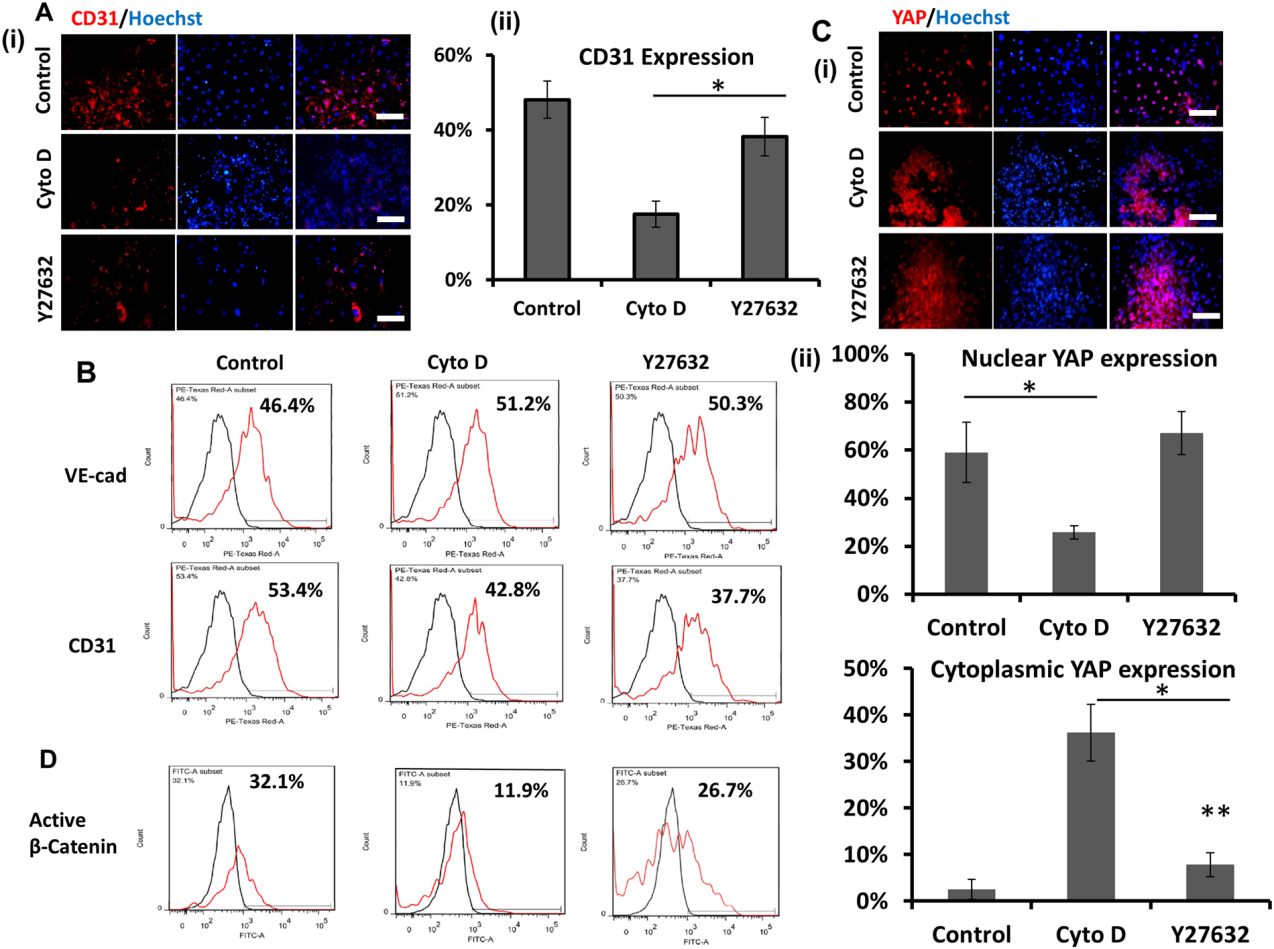
The effects of Cytochalasin D (CytoD) and ROCK inhibitor Y27632 (ROCKi) on cardiovascular spheroids formation. (**A**) (i) Representative fluorescent images of CD31 expression for CytoD (5 µM) and Y27632 (10 µM) treatment for 5 days after 15 day-differentiation. Scale bar: 100 μm. (ii) The quantitative analysis of CD31 (by image analysis). (**B**) The expression of CD31 and VE-cadherin by flow cytometry. (**C**) (i) Representative fluorescent images of YAP localization for CytoD (5 µM) and Y27632 (10 µM) treatment; (ii) The quantitative analysis of nuclear and cytoplasmic YAP expression (by image analysis). * and **indicate *p* < 0.05. (**D**) The expression of active β-catenin by flow cytometry. Black line: negative control; Red line: marker of interest.

Since the spheroid culture involves contractility effects and possible YAP localization, different small molecules including Cyto D, Y27632, Dasatinib, and LPA were used to treat the culture. First, the day 18 cardiac spheres were treatment with ROCK inhibitor Y27632^44^ or Cytochalasin D (Cyto D) for 5 days. After Cyto D treatment, the spheres significantly shrank and became more compact, whereas no difference in the shape of spheres for Y27632 treatments was observed compared to the control (Supplementary Fig. S7A). F-actin staining showed that Cyto D treatments caused disorganized cytoskeleton compared to the intact F-actin structures for Y27632 condition and control (Supplementary Fig. S7B). CD31 expression was decreased for Y27632 and Cyto D treatments (from 53.4% to 37.7% or 42.8% respectively) (Fig. 6A,B). The YAP localization and active β-catenin expression were also evaluated after the treatments. It was found that Cyto D treatment caused more cytoplasmic YAP and Y27632 slightly increased cytoplasmic YAP (Fig. 6C). The treatments also downregulated (from 32.1% to 11.9% for Cyto D treatment and to 26.7% for Y27632 treatment) the expression of transcription-active β-catenin, a key protein involved in Wnt/β-catenin signaling (Fig. 6D and Supplementary Fig. S7B). These results indicate that cytoskeleton remodeling could influence Wnt/β-catenin and YAP pathways and modulate cardiovascular patterning.

Since Cyto D and Y27632 are indirect YAP modulators and can cause other cellular events besides YAP re-localization, Dasatinib and LPA treatments were performed (Supplementary Fig. S8). Dasatinib is a direct inhibitor for nuclear YAP localization^45^, while LPA activates nuclear YAP^46^. Flow cytometry analysis showed the reduced VE-cadherin expression with Dasatinib treatment. But LPA treatment also decreased the level of VE-cadherin. Nkx2.5 expression was slightly increased for Dasatinib treatment, but decreased for LPA treatment compared to the control.

### The role of MMPs and cell-cell communications

**Figure 7:**
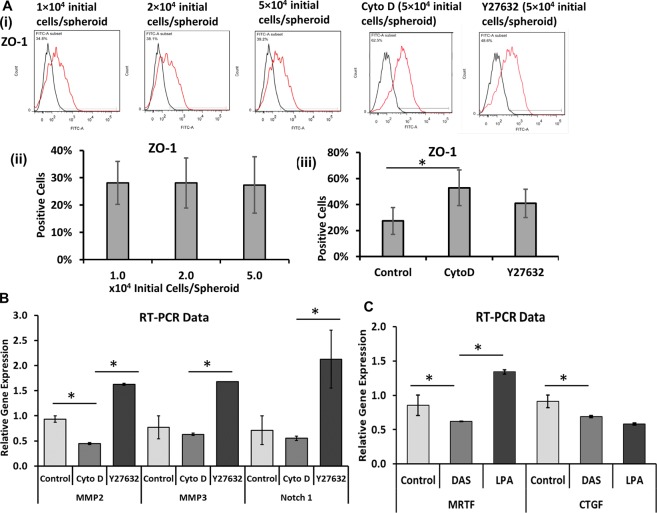
Characterizations of tight junction protein, cell-cell communication, and matrix remodeling proteins. (**A**) (i) Flow cytometry histograms of tight junction protein ZO-1. Black line: negative control; Red line: marker of interest. (ii) Quantification of ZO-1 expression by flow cytometry (n = 3) for different seeding densities, and for (iii) CytoD and Y27632 treated cells seeded with 5.0 × 10^4^ initial cells/spheroid in each well of 96-well plate. (**B**) RT-PCR analysis of MMP2, MMP3, and Notch-1 gene expression for CytoD and Y27632 treatments. (**C**) RT-PCR analysis of MRTF and CTGF gene expression for Dasatinib and Lysophosphatidic acid (LPA) treatments. *Indicates *p* < 0.05.

Zonula occludens-1 (ZO-1), the intercalated disk-related protein, has been reported to generate coupling between myocytes in association with Connexin 43 and remodel the cardiac gap junctions^47,48^. In our study, ZO-1 expression was similar for different seeding densities (e.g., 27.3 ± 10.3% for 5 × 10^4^/well), but increased to 40.9 ± 10.9% for Y27632 treatment and 52.9 ± 13.7% for Cyto D treatment (Fig. 7A). The NG2 expression (a marker for pericytes) was minimal (<4%) (data not shown). Due to the importance of matrix remodeling and cell-cell communications, the gene expression of MMP2, MMP3^49,50^, and Notch-1^51^ was analyzed by RT-PCR for the treatments with Cyto D and Y27632 (Fig. 7B). Cyto D treatment showed a reduction in MMP2 expression (0.45 ± 0.02 vs. 0.94 ± 0.06), but the MMP3 and Notch-1 expression was comparable to the control. Y27632 treatment increased the MMP2 (1.63 ± 0.02 vs. 0.94 ± 0.06), MMP3 (1.68 ± 0.00 vs. 0.77 ± 0.23), and Notch-1 (2.13 ± 0.58 vs. 0.71 ± 0.29) expression. Taking together, these data demonstrate that ROCK inhibitor Y27632 and Cyto D treatments could impact cell-cell and cell-matrix interactions during cardiovascular spheroid patterning.

MRTF, the transcription factor mediated by RhoGTPases, and the downstream YAP target gene CTGF were determined under Dasatinib (DAS) and LPA treatments (Fig. 7C). DAS reduced MRTF expression compared to the control (0.62 ± 0.00 vs. 0.86 ± 0.15), as well as reducing CTGF expression (0.69 ± 0.02 vs. 0.91 ± 0.09). LPA treatment increased MRTF expression compared to the control (1.34 ± 0.03 vs. 0.86 ± 0.15), but did not increase CTGF expression (0.58 ± 0.02 vs. 0.91 ± 0.09).

## Discussions

During cardiovascular differentiation of hPSCs, numerous factors such as cell density, substrate compliance, and paracrine factors can influence the patterning of cardiovascular progenitors into cardiac or vascular cells^52,53^. In this study, the influences of cell density and the corresponding YAP signaling activity on populations of cardiovascular mesoderm were investigated. Since different cell lines may require different levels of Wnt signaling for generating cardiac lineage^53^, CHIR99021 concentration and treatment time were firstly examined. Results from our study indicate that cardiomyocytes differentiation was optimal when the iPSK3 cells were treated with 10 μM CHIR99021 for 48 hours.

The confluency of culture before differentiation, the size of the undifferentiated hPSC aggregates, and the size of embryoid bodies (EBs) are critical parameters for efficient cardiac differentiation^19,53^. As the cell density increases, the cell-cell interactions and the associated paracrine factors affect the differentiation. For example, differential activity of glycogen synthase kinase 3 was suggested in suspension versus monolayer systems^54^. Our results indicate higher expression of α-actinin in 3D spheroid culture and the higher ratios of sarcomeric striations and Z-line sarcomeres than 2D culture. Other measurements for cardiac maturation need to be further developed in our future study. For example, ratios of MYH6/MYH7 or MYL7/MYL2 can be used to indicate cardiomyocyte maturation and other properties such as calcium signaling need to be characterized^20^. 3D spheroid cultures that are in favor of aerobic glycolysis may experience metabolic shift compared to 2D cultures^55^, resulting in strong anaerobic phenotype. Our results showed that there existed a co-expression of vascular markers CD31 and VE-cadherin in the spheroids, suggesting that the 3D suspension culture may support both cardiac and vascular cell generation. Another study reported that downregulation of LGR5 shifts the lineage commitment of hPSCs from cardiomyocytes to endothelial cells^56^. Formation of definitive mesoderm and the cardiac mesoderm by paracrine factors was reported to depend on the size of the aggregates (indicates spatial cell density)^57^. Different size of aggregates leads to either cardiac or endothelial cell differentiation in microwells^58^. Also, the paracrine effect was reported to produce a significantly higher frequency of hematopoietic cells in EB culture^59^. These observations suggest that the cultures of different cell densities in 3D systems might support differentiation towards various lineages in the cardiovascular mesoderm other than cardiomyocytes. During our investigation, different seeding densities gave rise to spheroids of comparable size at a late stage, but only the higher density of the cells supported the differentiation of cardiac cells and vascular cells, while the cultures with lower density did not express cardiac markers.

One potential mechanism that is consistent with our data is a change in YAP signaling. Hippo/YAP signaling has been explored for regulation of cardiomyocyte proliferation and the heart size^33^. Active YAP can enter the nucleus and bind with lineage-specific markers and thereby influence the differentiation, but YAP is sensitive to cell density and tissue structure^31,60^. Recent studies shed light on the fact that the fate decisions of cardiovascular progenitors depend on the shuttling of YAP between nucleus and cytoplasm^35,61^. YAP localization can modulate the progenitor commitment to either cardiomyocytes or endothelial cells. Our analysis on the localization of YAP indicates that higher density keeps YAP cytoplasmic whereas the lower cell density promotes nuclear YAP. The results suggest that YAP re-localization, possibly caused by a global change in cell biology downstream of the modulations in 3D culture, might influence the balance of cardiac and vascular populations generated from cardiovascular spheroids.

Specifically, the treatments of the cells with actin depolymerizing agent Cytochalasin D, ROCK inhibitor Y27632, Dasatinib, and LPA were used to control YAP localization in our study^38,46^. Actin stress fibers were reported to reduce YAP phosphorylation and promote nuclear YAP accumulation; thus, disruption of stress fibers using cytochalasin D results in the cytoplasmic YAP localization^39^. ROCK inhibition using Y27632 controls the assembly of actin cytoskeleton and cell contractibility by phosphorylation of myosin light chain phosphatase etc.^44^. Over-active ROCK can inhibit actin cytoskeleton disassembly and increase the number of actin filaments. Dasatinib directly inhibits nuclear YAP and thus the YAP-target genes. For comparison, LPA was included to promote nuclear YAP. From our results, Dasatinib indeed reduced MRTF, the transcription factor mediated by RhoGTPases, and YAP-target gene CTGF. LPA upregulated MRTF, as expected, but did not increase CTGF. Both Cyto D and Y27632 reduced the expression of the vascular marker. Dasatinib also reduced VE-cadherin expression, but LPA failed to increase the level of VE-cadherin. These results indicate that both density and YAP localization can impact the differentiation. It was also realized that small molecule treatments may alter a large number of transcripts. Therefore, a thorough analysis of the treatment consequences would require the analysis of transcriptome using genomics tool.

Our previous work focused on the interaction of YAP with Wnt/β-catenin signaling and the corresponding impacts on neural patterning of hPSCs^37,38^. In this study, the impact of YAP localization on the active β-catenin expression during cardiovascular patterning of hiPSCs was investigated. The treatment with Y27632 reduced the expression of active β-catenin slightly, but the Cyto D treatment significantly reduced the active β-catenin expression. The results from this study suggest that YAP localization and the corresponding effects on Wnt signaling seem to contribute to the cell population distribution. It needs to be noted that the influence of Cyto D treatment on YAP localization is an indirect method. The direct method to target Hippo/YAP should be preferably performed with specific reagents like shRNA or siRNA^31^.

Besides canonical Wnt signaling, high-density culture with cytoplasmic YAP was reported to upregulate Notch-3 expression, while nuclear YAP inhibited Notch signaling^62^. Our results show that Cyto D treatment (disrupt actin filaments) induced more cytoplasmic YAP, but did not upregulate Notch-1, while Y27632 treatment (inhibits cellular contractility by inhibiting ROCK) slightly increased cytoplasmic YAP (inhibiting Hippo signaling) and significantly upregulated Notch-1 gene expression. Hippo/YAP signaling (with nuclear YAP) was reported to inhibit Notch signaling by inhibiting the expression of Jag1 that is activated by YAP^63^. The difference in response to Cyto D and Y27632 treatments in our study may indicate that appropriate YAP signaling level is required for the upregulation of Notch signaling.

Our results indicate that the cells with Y27632 treatment expressed higher levels of MMP-2 and MMP-3 gene expression, which may be beneficial for future study on angiogenesis and blood vessels sprouting. Pathologically, increased MMP-2 expression was observed after myocardial infarction (MI), which increases left ventricle rupture and delays post-MI remolding^50^. An elevated level of MMP-3, a stromelysin subtype, acts as an upstream regulator in the activation of other MMPs and is associated with increased MI risk^50^. In *in vitro* culture, MMP2/3 promotes matrix remodeling and thus the cell migration^64^. ROCK inhibitors have been suggested as an attractive therapeutic target for reducing cardiovascular diseases^44^.

It is realized that evidence for CD31^+^ cells alone in our study does not indicate the established conditions allowing for vascularization. The blood vessel structure requires the coordination of endothelial cells, pericytes, and smooth muscle cells, which needs additional great efforts to understand heterotypic cell-cell interactions. Taken together, the results from this study have the implications in generation of 3D cardiovascular models from hPSCs.

## Conclusions

This study investigates the underlying effects of cell density and possible YAP signaling on the patterning of cardiovascular precursors from hiPSCs in 3D spheroids. The results indicate that the cell densities can impact the cell-cell interactions and the contractility of 3D spheroids. The associated YAP signaling and the influences on Wnt and Notch signaling activities in the cardiovascular spheroids may affect the patterning of cardiac and vascular cells. Future work would put more focus on the modulation of cell signaling and cellular composition to produce vascularized cardiac tissues or organoids that can mimic heart and blood vessels regeneration efficiently.

## Methods

### Undifferentiated hiPSC culture

Human iPSK3 cells were derived from human foreskin fibroblasts transfected with plasmid DNA encoding reprogramming factors OCT4, NANOG, SOX2 and LIN28 (kindly provided by Dr. Stephen Duncan, Medical College of Wisconsin, and Dr. David Gilbert, Department of Biological Sciences of Florida State University)^65,66^. Human iPSK3 cells were maintained in mTeSR serum-free medium (StemCell Technologies, Inc., Vancouver, Canada) on 6-well plates coated with growth factor-reduced Geltrex (Life Technologies, Carlsbad, CA). The cells were passaged by Accutase dissociation every 5–6 days and seeded at 1 × 10^6^ cells per well of 6-well plate in the presence of 10 μM Y27632 (Sigma) for the first 24 hours^38,67,68^.

### Cardiac differentiation, cardiac spheroid, and cardiovascular spheroid formation

#### Monolayer-based cardiac differentiation and cardiac enrichment by sphere formation

Human iPSK3 cells were seeded on 24-well plates coated with Geltrex (Life Technologies) at a cell density of 4 × 10^5^ cells/well. Cells were maintained in mTeSR serum-free medium with 10 µM ROCK inhibitor (ROCKi) Y27632 (Sigma) for the first day and in mTeSR medium only for another four days. The cardiomyocytes differentiation was induced by modulating Wnt pathways with small molecules CHIR99021 (a Wnt activator) and IWP4 (a Wnt inhibitor) (Giwi protocol)^11^. To optimize the protocol, the treatment conditions of CHIR99021 (StemCell Technologies, Inc.) were investigated. Briefly, cells were cultivated in RPMI plus 2% B27 serum-free supplement minus insulin medium (Life Technologies) with 5 µM or 10 µM CHIR99021 (day 0) for 24 hours or 48 hours and then CHIR99021 was withdrawn from the medium. After another two days, the cells were then cultivated in the medium with 5 µM IWP4 (Stemgent) for two days. At day 6 or 7, the medium was changed to RRMI plus 2% B27 and beating cells were observed at day 12–14. Based on the analysis of expression of cardiac markers, the optimized condition for CHIR99021 induction was 10 µM and 48 hours treatment.

Cardiac differentiation was also induced with growth factors. Undifferentiated iPSK3 cells were treated with 100 ng/mL recombinant human activin A (R&D Systems) in RPMI/B27-insulin medium (day 0). On day 1, the cells were maintained in RPMI/B27-insulin medium supplemented with 10 ng/mL recombinant human bone morphogenetic protein 4 (BMP-4; R&D Systems). Four days later (i.e., on day 5), the medium was switched to RPMI/B27 and subsequently the cells were fed every alternative day with this medium until harvest^7,14^. To enhance cardiomyocyte differentiation, the day 15 cells in monolayer (2D) were harvested and cultured in Ultra-Low Attachment (ULA) 24-well plate (Corning) for cardiac sphere formation.

#### Formation of cardiovascular spheroids

The cardiac and cardiovascular spheroids were generated by seeding human iPSK3 cells into ULA 24-well or 96-well plates and induced for differentiation using the optimized protocol from monolayer culture. To evaluate the effects of cell density on spheroid formation, three different densities 1 × 10^4^ cells/well, 2 × 10^4^ cells/well, and 5 × 10^4^ cells/well were compared for spheroids cultured in 96-well plate, in which each well contained one spheroid. To evaluate the effects of Geltrex and ROCK inhibitor Y27632, iPSK3 cells were cultivated in 1% Geltrex or 10% Geltrex, and with or without 10 µM ROCK inhibitor Y27632 for 7 days in the ULA 24-well plates.

### LIVE/DEAD assay

Live/Dead^®^ staining kit (Molecular Probes) was used to assess cell viability. Immediately after harvesting, the cells were incubated in DMEM-F12 containing 1 μM calcein AM and 2 μM ethidium homodimer I for 30 min. The samples were then washed and imaged under a fluorescent microscope (Olympus IX70, Melville, NY).

### Immunocytochemistry

Briefly, the samples were fixed with 4% paraformaldehyde (PFA) and permeabilized with 0.2–0.5% Triton X-100 for intracellular markers. The samples were then blocked and incubated with various mouse or rabbit primary antibodies (Supplementary Table S1). After washing, the cells were incubated with the corresponding secondary antibody: Alexa Fluor® 488 goat anti-Mouse IgG or Alexa Fluor® 594 goat anti-Rabbit IgG (Life Technologies). The samples were stained with Hoechst 33342 and visualized using a fluorescent microscope (Olympus IX70, Melville, NY) or a confocal microscope (Zeiss LSM 880). The proportion of positive cells was calculated based on the area of marker of interest normalized to the nuclei using ImageJ analysis, indicating the relative expression among different conditions.

*Analysis of YAP expression*: nuclear and cytoplasmic YAP was determined based on the expression pattern of image analysis using ImageJ software. The Hoechst expression of each image was superimposed on YAP image. Then the nuclear YAP was counted and compared with the total Hoechst to obtain the relative expression^38,69^.

### Aggregate size distribution and analysis of sarcomere length and Z-line

The images of spheroids were captured over the culture time by a phase contrast microscopy. The captured images were converted to binary images using ImageJ software (http://rsb.info.nih.gov/ij) and analyzed with the “particle analysis tool”. Through particle analysis in ImageJ software, the Feret’s diameter of each aggregate in the images can be calculated, which provided the size distribution of the aggregates.

To evaluate z-line sarcomere organization of different conditions, about 150–600 of 2D and 3D α-actinin^+^ cardiomyocytes were “scored” for their relative levels of z-line sarcomere organization. The length and width of z-line sarcomere were quantified in ImageJ software using images of α-actinin staining from n = 18 to 20 cells per condition.

### Flow cytometry

To quantify the levels of various marker expression, the cells were harvested by trypsinization and analyzed by flow cytometry. Briefly, 1 × 10^6^ cells per sample were fixed with 4% PFA and washed with staining buffer (2% fetal bovine serum in phosphate buffer saline). The cells were permeabilized with 100% cold methanol for intracellular markers, blocked, and then incubated with primary antibodies against α-actinin, active β-catenin, Nkx2.5, CD31, Zonula occludin 1 (ZO-1), NG2, and VE-cadherin followed by the corresponding secondary antibody (Supplementary Table S1). The cells were acquired with BD FACSCanto™ II flow cytometer (Becton Dickinson) and analyzed against isotype controls using FlowJo software.

### Treatments with ROCKi Y27632, Cytochalasin D, Dasatinib, and LPA

To evaluate the effect of stress fiber on cardiovascular spheroid composition, the day 18 cardiac spheres (seeded with 5 × 10^4^ initial cells/spheroids) were treatment with 1 µM or 10 µM ROCK inhibitor Y27632 and 1 µM or 5 µM Cytochalasin D (CytoD, an inhibitor of actin polymerization, Sigma) for 5 days in the ULA 96-well plates. The samples were then harvested for immunostaining, Phalloidin staining, and mRNA isolation.

In addition, Dasatinib (300 nM, a direct inhibitor for nuclear YAP localization, Sigma)^45^ and LPA (0.5–5 µM, an inducer of actin polymerization, Sigma)^46^ were used to treat day 14 cardiac spheres for 3 days in the ULA 96-well plates. The samples were then harvested for immunostaining and mRNA isolation.

### Reverse transcription polymerase chain reaction (RT-PCR) analysis

Total RNA was isolated from different samples using the RNeasy Mini Kit (Qiagen, Valencia, CA) according to the manufacturer’s protocol followed by the treatment of DNA-Free RNA Kit (Zymo, Irvine, CA)^69^. Reverse transcription was carried out using 2 μg of total RNA, anchored oligo-dT primers (Operon, Huntsville, AL), and Superscript III (Invitrogen, Carlsbad, CA) (according to the protocol of the manufacturer). Primers specific for target genes (Supplementary Table S2) were designed using the software Oligo Explorer 1.2 (Genelink, Hawthorne, NY). The gene β-actin was used as an endogenous control for normalization of expression levels. Real-time RT-PCR reactions were performed on an ABI7500 instrument (Applied Biosystems, Foster City, CA), using SYBR1 Green PCR Master Mix (Applied Biosystems). The amplification reactions were performed as follows: 2 min at 50 °C, 10 min at 95 °C, and 40 cycles of 95 °C for 15 sec and 55 °C for 30 sec, and 68 °C for 30 sec. Fold variation in gene expression was quantified by means of the comparative Ct method: ${2}^{-({C}_{ttreatment}-{C}_{tcontrol})}$ which is based on the comparison of expression of the target gene (normalized to the endogenous control β-actin) between the treated spheroids and the no-treatment spheroid control.

### Statistical analysis

Each experiment was carried out at least three times. The representative experiments were presented and the results were expressed as [mean ± standard deviation]. To assess the statistical significance, one-way ANOVA followed by Fisher’s LSD post hoc tests were performed. A *p*-value < 0.05 was considered statistically significant.

## Supplementary information

Supplementary Materials

Monolayer beating cells

Sphere beating cells
