# Supplementary material for: Cell population balance of cardiovascular spheroids derived from human induced pluripotent stem cells
Source: Sci Rep. 2019 Feb 4;9:1295. doi: 10.1038/s41598-018-37686-1 (PMC6362271; doi:10.1038/s41598-018-37686-1)
Supplement: Supplementary file 1 — Supplementary Materials [file 41598_2018_37686_MOESM1_ESM.docx]

**Supplementary Materials**

**Cell population balance of cardiovascular spheroids derived from human induced pluripotent stem cells**

Yuanwei Yan^#, a^, Julie Bejoy^#^, Junfei Xia ^b^, Kyle Griffin, Jingjiao Guan, Yan Li*

Department of Chemical and Biomedical Engineering; FAMU-FSU College of Engineering; Florida State University; Tallahassee, FL USA

**Supplementary Figure S1. The Schematic illustration of cardio differentiation from hiPSCs.** (A) The Giwi protocol using CHIR99021 and IWP4; (B) The A100/B10 protocol using Activin A and BMP-4.


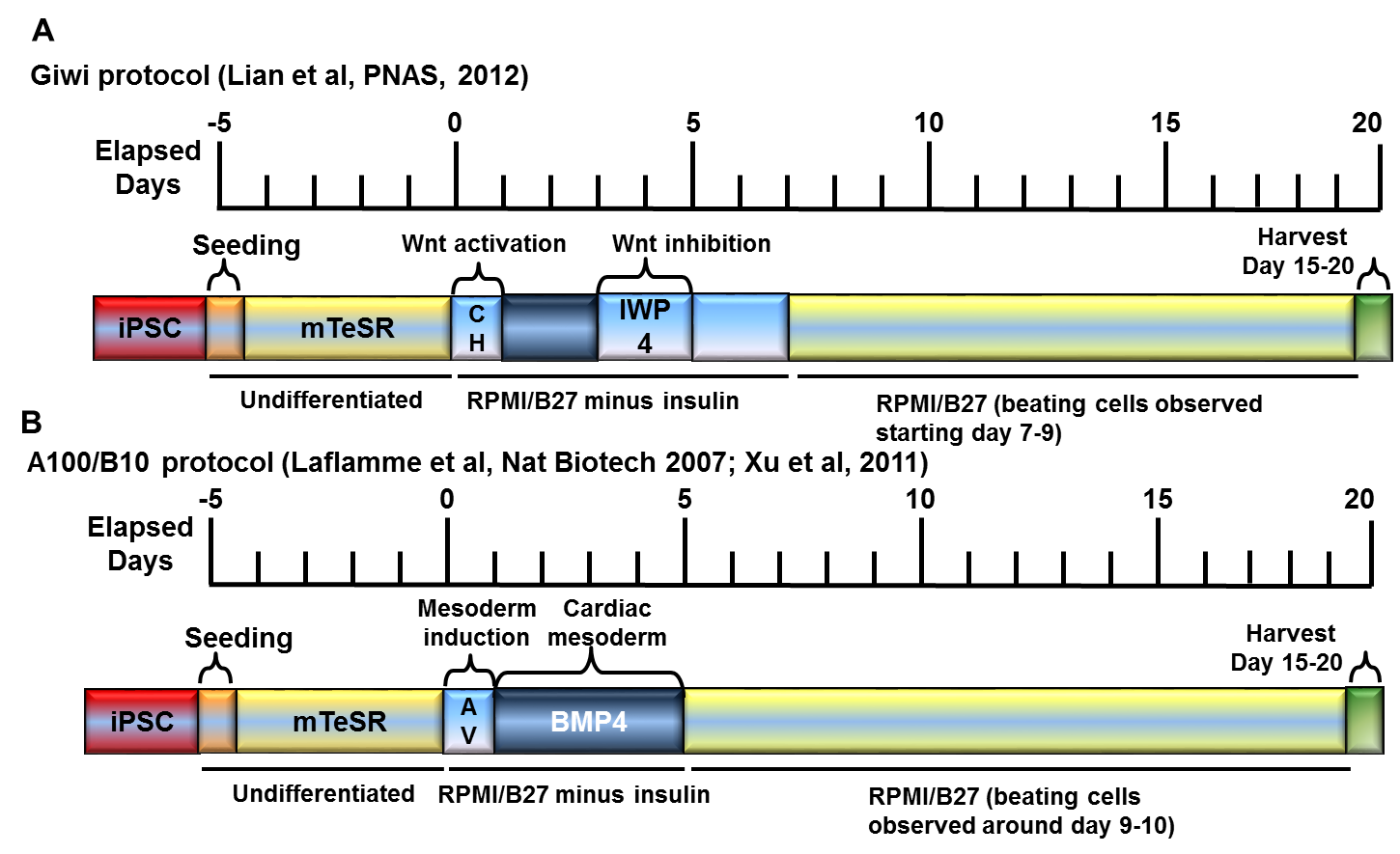


**Supplementary Figure S2.** **Representative fluorescent images of cardiac markers Nkx2.5 and α-actinin for replated cardiac spheres.** (A) Day 20 and (B) day 35. Scale bar: 40 μm. (C) Negative control for Nkx2.5. Scale bar: 100 μm.

**
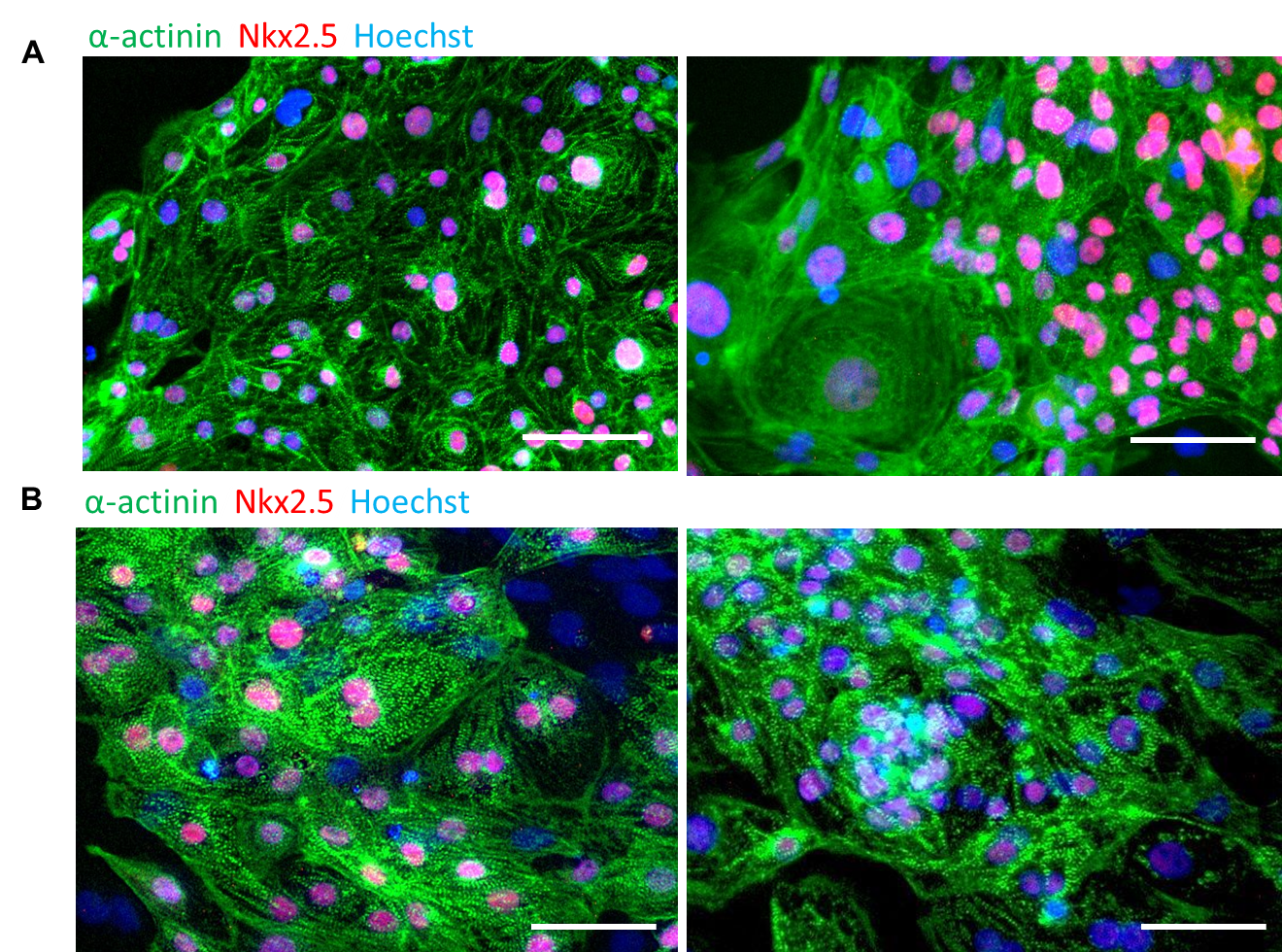
**

**
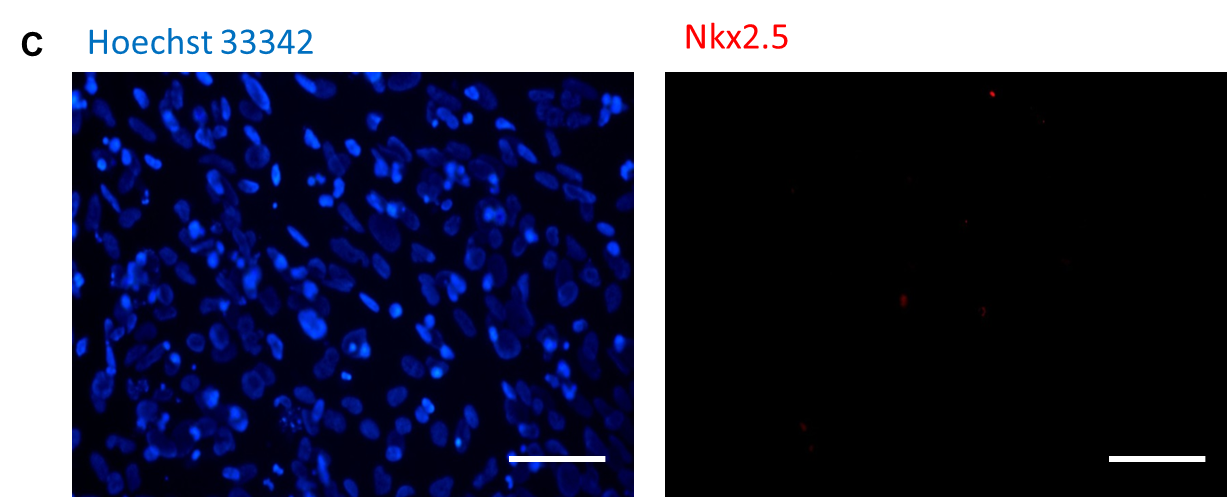
**

**Supplementary Figure S3. Measurements of beating activity for cardiovascular spheres at different days.** (A) The beating frequency (beats per minute); (B) The proportion of beating spheres.


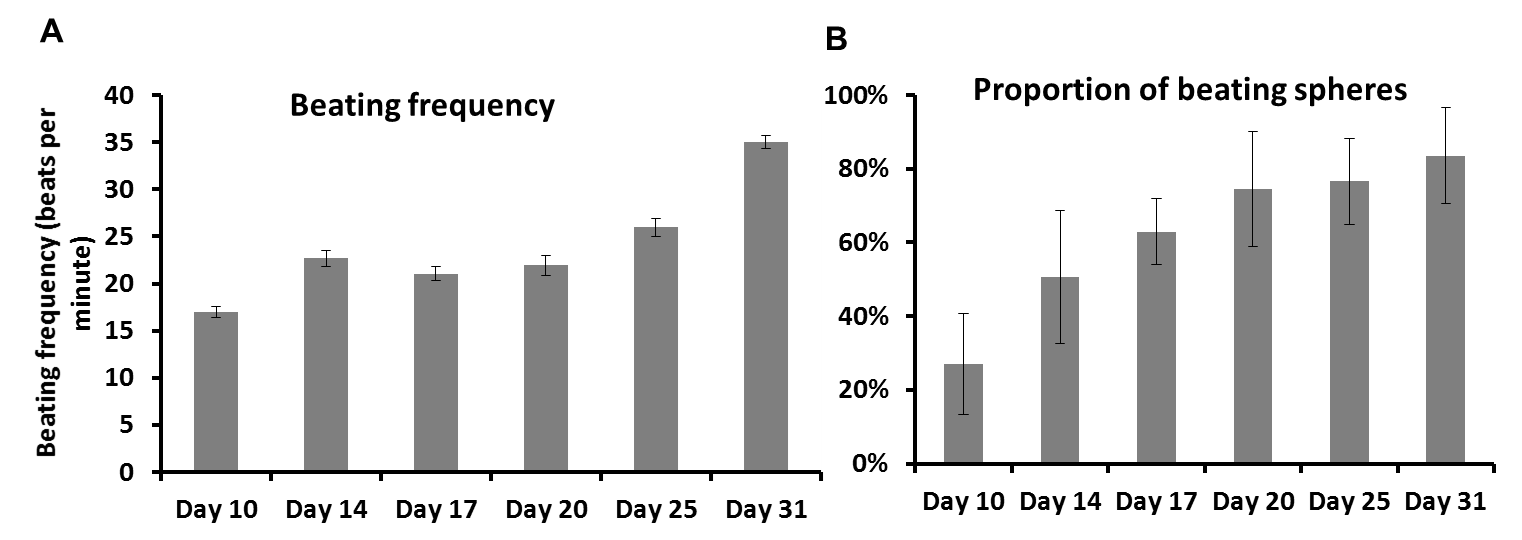


**Supplementary Figure S4. The cardiovascular spheroid formation for different seeding densities.** (A) Phase contrast images of cardiovascular spheroids for different days. Scale bar: 200 μm. (B) The average diameter of spheres for different conditions.


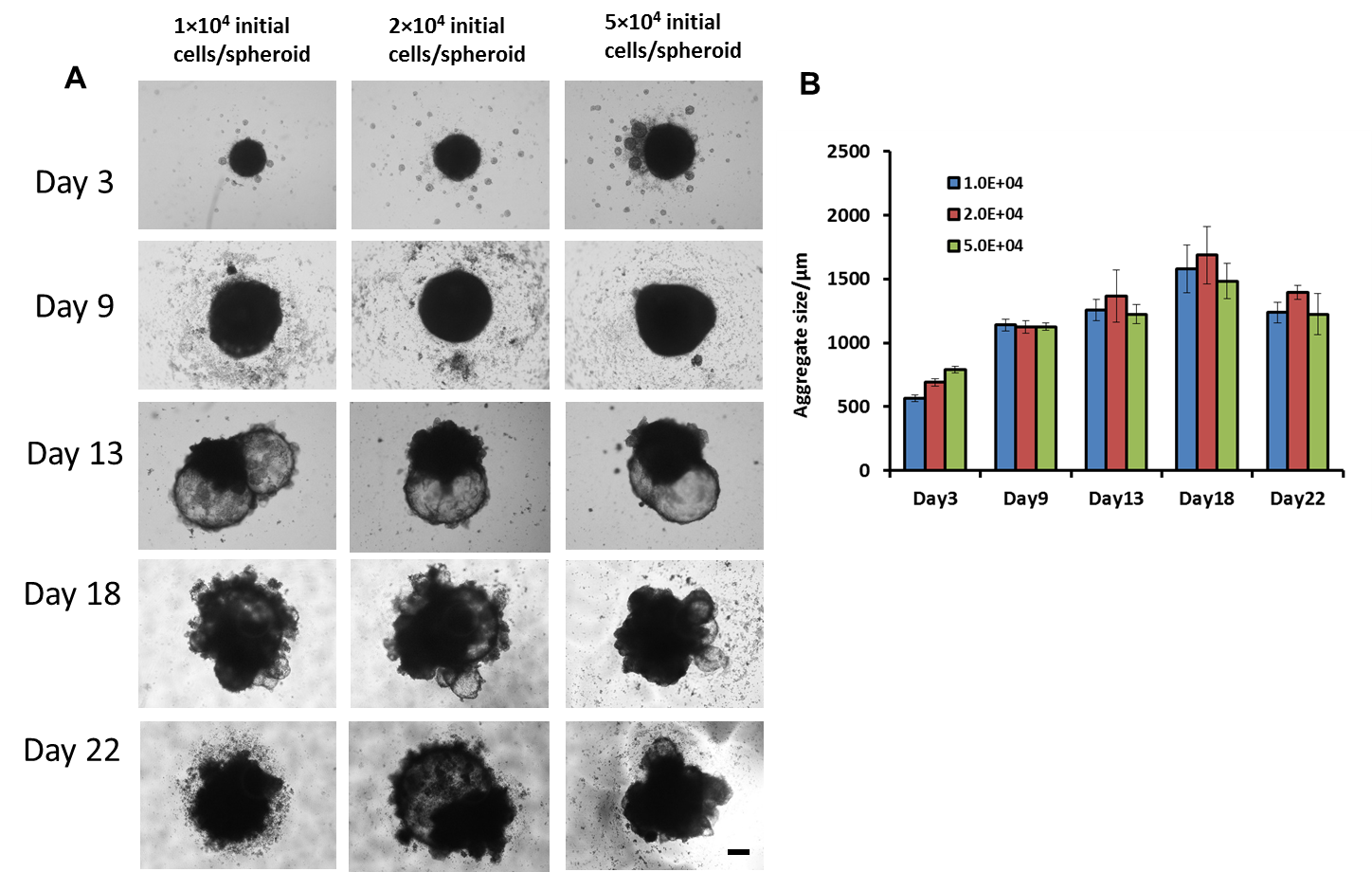


**Supplementary Figure S5. The localization of CD31 in spheroids examined using confocal microscopy.** The staining was performed on day 15 spheroids. Scale bar: 100 μm.


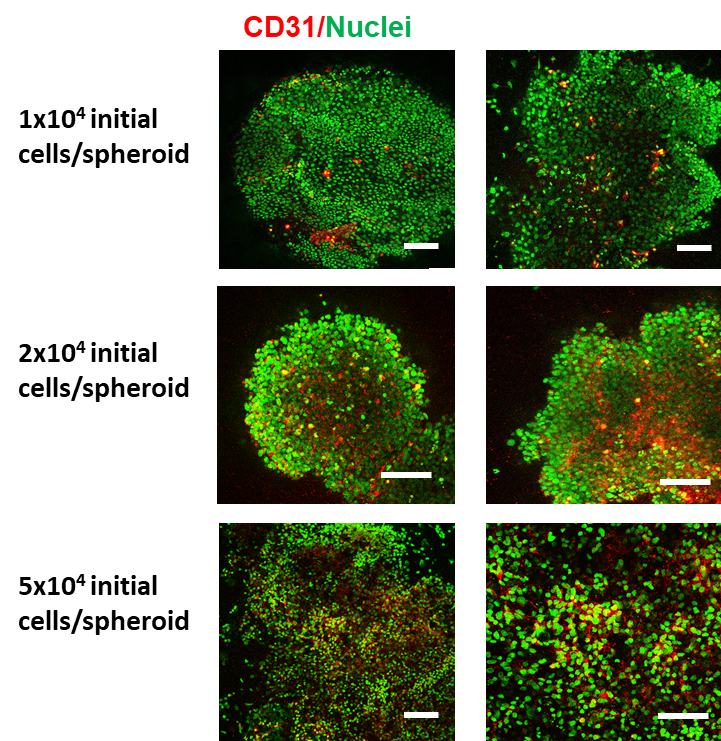


**Supplementary Figure S6. The effects of Geltrex and ROCK inhibitor** **Y27632 (ROCK i) on cardiac spheroid formation.** (A) (i) The phase contrast images of cardiac spheroid formation for different conditions from day 1 to day 7. 1% Geltrex leads to high ECM protein concentration in the culture medium, while 10% Geltrex may form the hydrogels (at 37^o^C). Different Geltrex concentrations were evaluated to see how hydrogels affect 3D aggregation. (ii) The average diameter of cardio sphere. (B) The cell viability of spheres after the incubation by Live/Dead assay. Scale bar: 100 μm.


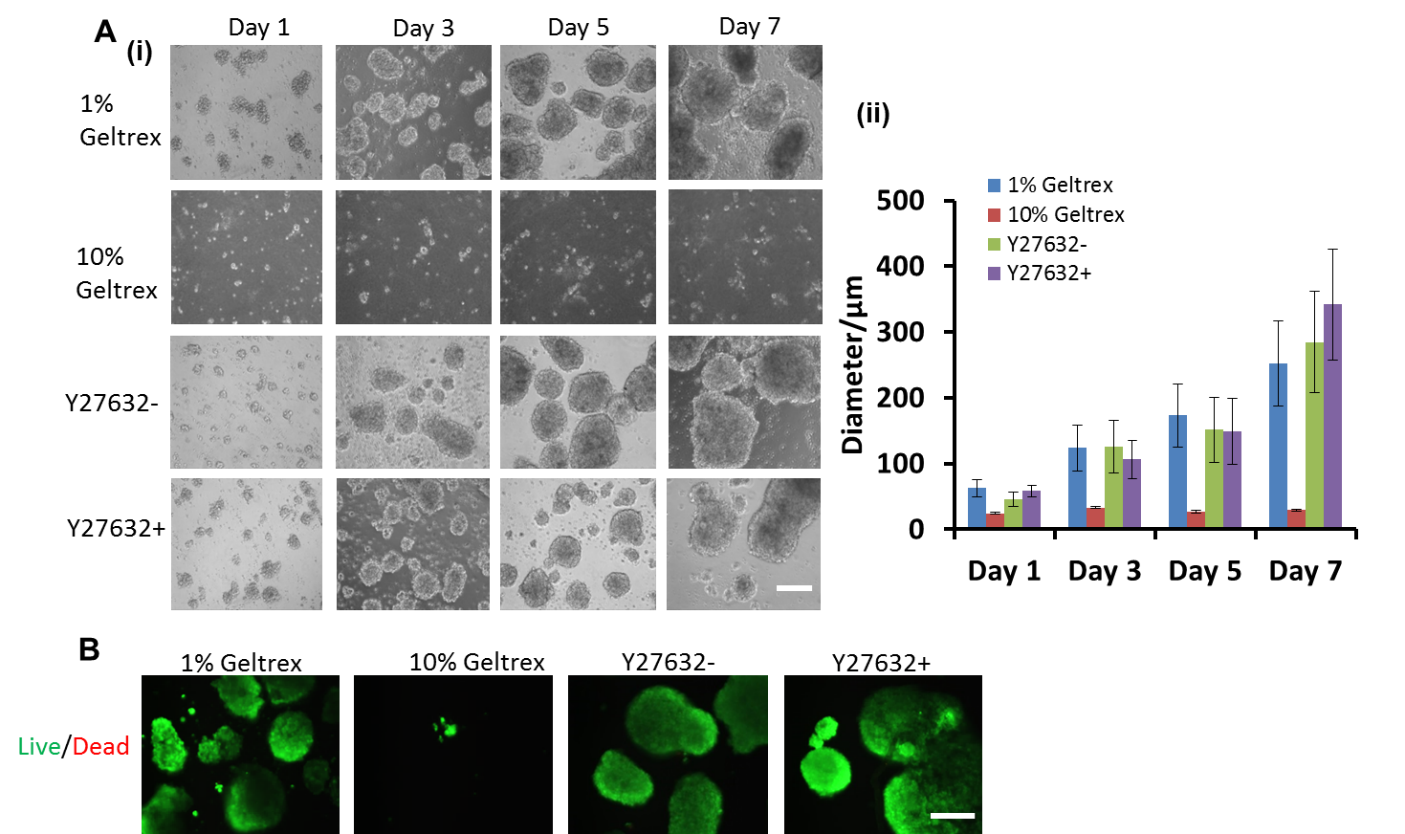


**Supplementary Figure S7. The effects of Cytochalasin D (CytoD) and ROCKi Y27632 on cardiovascular spheroid formation.** (A) The phase contrast images of cardiovascular spheres after the treatments. (B) Representative fluorescent images of F-actin and active-β-catenin for CytoD (5 µM) and ROCKi Y27632 (10 µM) treatment for 5 days. Scale bar: 100 μm.


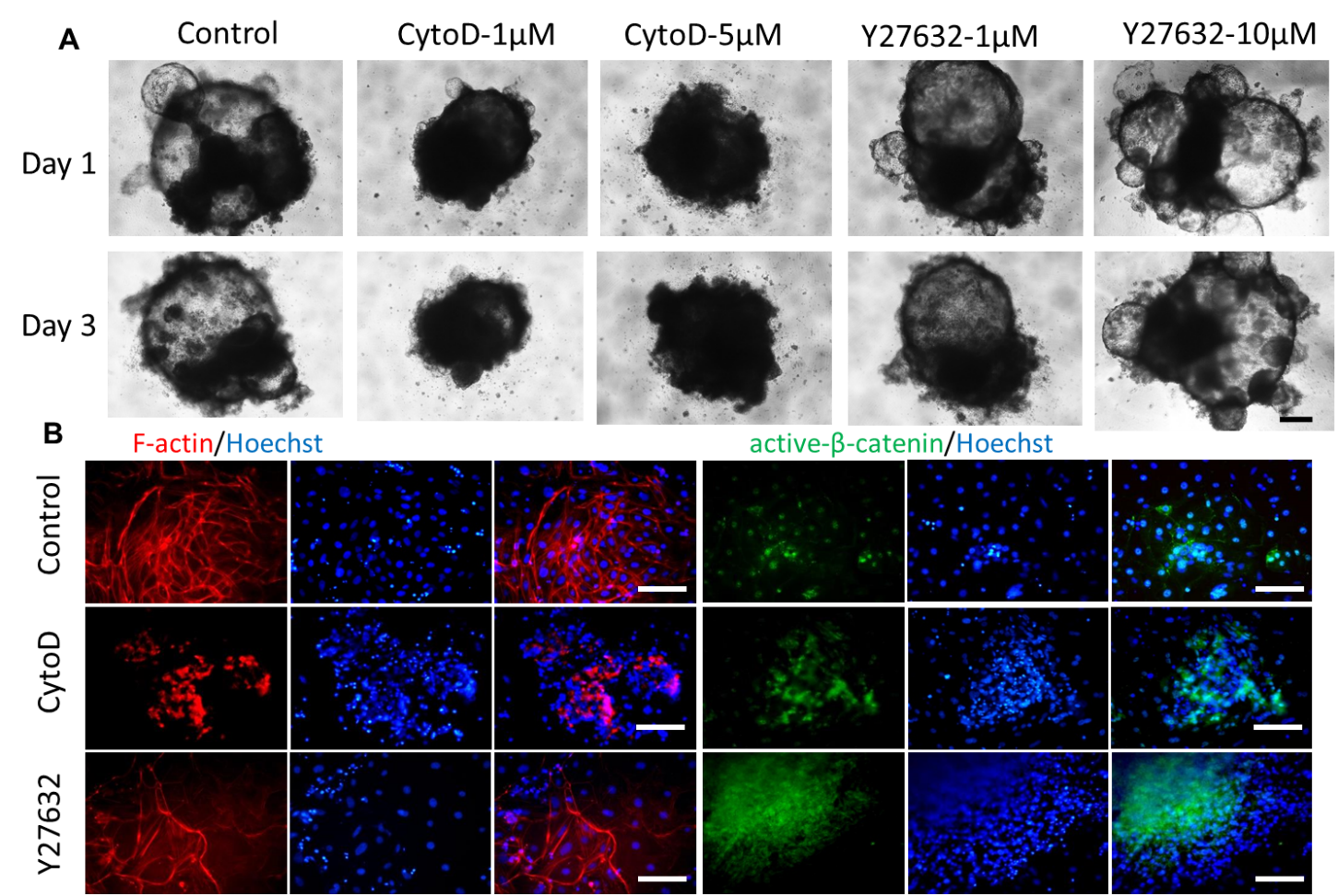


**Supplementary Figure S8.** **Effects of Dasatinib and Lysophosphatidic acid (LPA) treatments on VE-cadherin and Nkx2.5 expression.** (A) VE-Cadherin; (B) Nkx2.5. Black line: negative control; Red line: marker of interest.


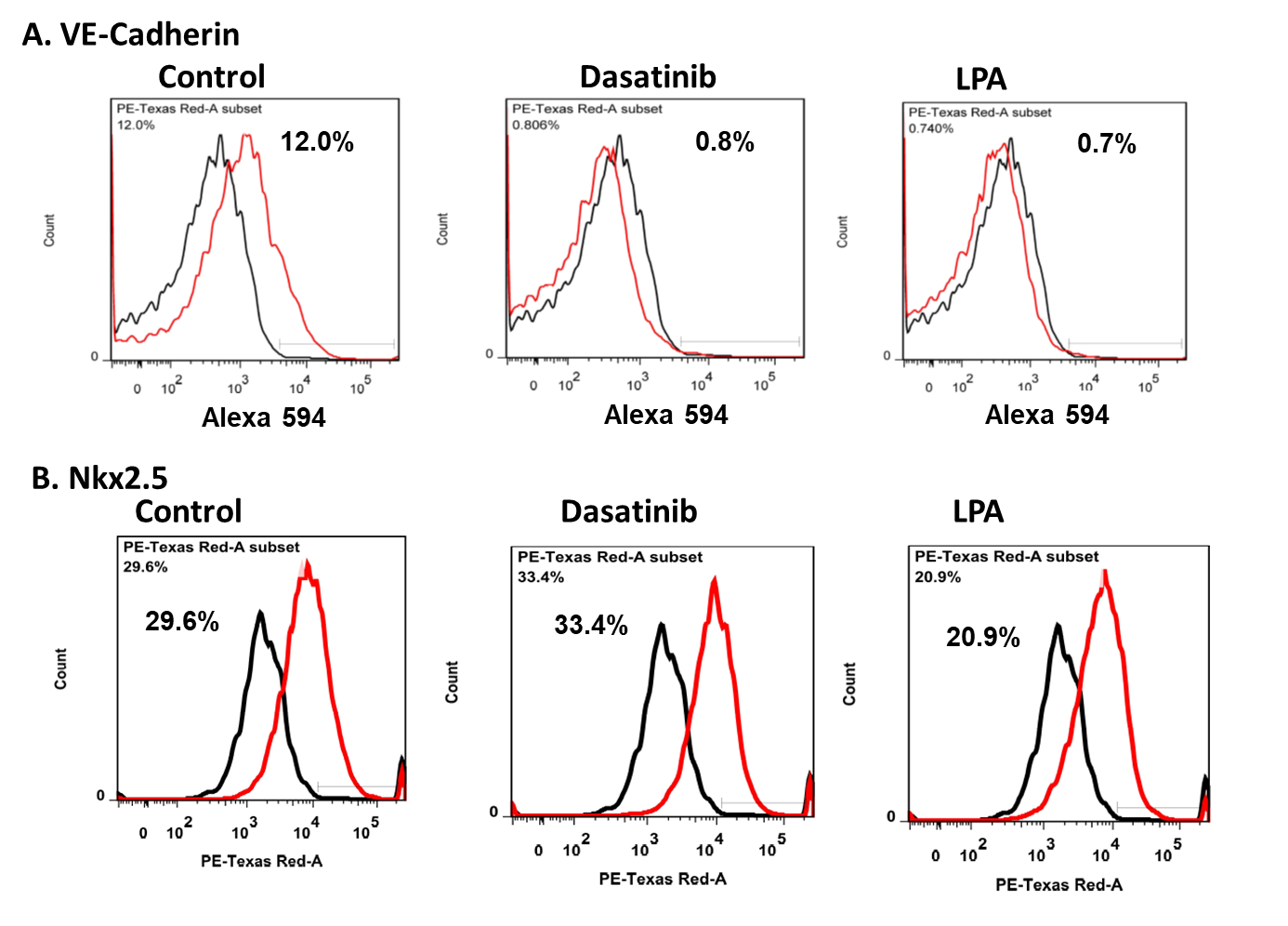


**Supplementary Video 1. Representative beating sheet in monolayer (2D) culture (day 20).**

**Supplementary Video 2. Representative beating cardiac spheroids in 3D culture (day 17).**

**Supplementary Table S1. A list of antibodies**

| **Cells** | **Primary Antibody** | **Origin/ Isotype** | **Supplier/ Cat#** | **Dilution** |
| --- | --- | --- | --- | --- |
| Cardiac cells | α-actinin | Mouse IgG1 | Sigma, A7811 | 1:800 |
|  | Nkx2.5 | Rabbit Polyclonal IgG | Santa Cruz, sc-14033 | 1:400 |
| Vascular cells | CD31 | Goat polyclonal IgG | Santa Cruz, sc-1506 | 1:200 |
|  | VE-cadherin | Goat polyclonal IgG | Santa Cruz, sc-6458 | 1:200 |
| Tight junctions | ZO-1 | Mouse IgG1 | ThermoFisher, 33-9100 | 1:100 |
| Pericytes | NG2 | Mouse IgG1 | Millipore, MAB5384 | 1:100 |
| Pathway | YAP | Rabbit Polyclonal IgG | Santa Cruz, sc-15407 | 1:100 |
|  | F-actin | Phalloidin 594 | Molecular Probes, A12381 | 1:100 |
|  | Active β-catenin | Mouse IgG1 | Millipore, 05-665 | 1:100 |
| Secondary | Alexa 488, goat anti-mouse IgG1 | - | Life Technologies,  A-21121 | 1:200 |
|  | Alexa 488, goat anti-rabbit IgG | - | Life Technologies,  A-11034 | 1:200 |
|  | Alexa 594, goat anti-rabbit IgG | - | Life Technologies,  A-11012 | 1:400 |
|  | Alexa 594, donkey anti-goat IgG | - | Life Technologies,  A-11058 | 1:400 |

**Supplementary Table S2. Primer sequence for target genes.**

| Gene | Forward primer 5' to 3' | Reverse primer 5' to 3' |
| --- | --- | --- |
| MMP2 | CATCGCTCAGATCCGTGGTG | GCATCAATCTTTTCCGGGAGC |
| MMP3 | CCATCTCTTCCTTCAGGCGT | ATGCCTCTTGGGTATCCAGC |
| Notch-1 | CACTGCTGCCCTCCCCGTTC | TTCAGGTGCCCGATGCCCAG |
| MRTFA | CCTTTCCCTCATTGACGACCT | ACAAAGTGCAATTCCGAGGTG |
| CTGF | AGCTGACCTGGAAGAGAACA | CAGGCACAGGTCTTGATGAA |
| β-actin | GTACTCCGTGTGGATCGGCG | AAGCATTTGCGGTGGACGATGG |
